# Photosynthetic apparatus performance of tomato seedlings grown under various combinations of LED illumination

**DOI:** 10.1371/journal.pone.0249373

**Published:** 2021-04-15

**Authors:** Ahmed F. Yousef, Muhammad M. Ali, Hafiz M. Rizwan, Shehu Abubakar Tadda, Hazem M. Kalaji, Hao Yang, Mohamed A. A. Ahmed, Jacek Wróbel, Yong Xu, Faxing Chen

**Affiliations:** 1 College of Horticulture, Fujian Agricultural and Forestry University, Fuzhou, China; 2 Department of Horticulture, College of Agriculture, University of Al-Azhar (branch Assiut), Assiut, Egypt; 3 Department of Crop Production and Protection, Faculty of Agriculture and Agric. Technology, Federal University, Dutsin-Ma, Katsina, Nigeria; 4 Department of Plant Physiology, Institute of Biology, Warsaw University of Life Sciences SGGW, Warsaw, Poland; 5 College of Mechanical and Electronic Engineering, Fujian Agriculture and Forestry University, Fuzhou, China; 6 Plant Production Department (Horticulture—Medicinal and Aromatic Plants), Faculty of Agriculture (Saba Basha), Alexandria University, Alexandria, Egypt; 7 Department of Bioengineering, West Pomeranian University of Technology in Szczecin, Szczecin, Poland; 8 Institute of Machine Learning and Intelligent Science, Fujian University of Technology, Fuzhou, China; United Arab Emirates University, UNITED ARAB EMIRATES

## Abstract

It is already known that the process of photosynthesis depends on the quality and intensity of light. However, the influence of the new light sources recently used in horticulture, known as Light Emitting Diodes (LEDs), on this process is not yet fully understood. Chlorophyll *a* fluorescence measurement has been widely used as a rapid, reliable, and noninvasive tool to study the efficiency of the photosystem II (PSII) and to evaluate plant responses to various environmental factors, including light intensity and quality. In this work, we tested the responses of the tomato photosynthetic apparatus to different light spectral qualities. Our results showed that the best performance of the photosynthetic apparatus was observed under a mixture of red and blue light (R7:B3) or a mixture of red, green and blue light (R3:G2:B5). This was demonstrated by the increase in the effective photochemical quantum yield of PSII (Y[II]), photochemical quenching (qP) and electron transport rate (ETR). On the other hand, the mixture of red and blue light with a high proportion of blue light led to an increase in non-photochemical quenching (NPQ). Our results can be used to improve the production of tomato plants under artificial light conditions. However, since we found that the responses of the photosynthetic apparatus of tomato plants to a particular light regime were cultivar-dependent and there was a weak correlation between the growth and photosynthetic parameters tested in this work, special attention should be paid in future research.

## 1 Introduction

Tomato is a strategic crop worldwide. To obtain healthy and productive tomato plants, a high-quality planting material is a necessity. Light (intensity, quality and duration) is one of the fundamental factors that determine the process of photosynthesis and plant growth [1]. Light also affects the content of primary and secondary metabolites in plants [2]. Under artificial growing conditions, lighting system can determine the cost and nutrient quality of plants [3, 4]. In recent years, many researches have proved that the Light Emitting Diodes (LEDs) are a suitable light source for lighting growth chambers and greenhouses [5–7]. However, the recommendations for the appropriate light for a particular plant are still under discussion and open researches.

Over the years, different light conditions have been considered to conduct the physiological studies related to photosynthesis process. A combination of different light spectral such as red, blue, and white of varying intensities has been considered an efficient and effective source for plants’ photosynthesis performance [5]. Moreover, several authors [7–9] found that the red light is important for development of photosynthetic apparatus in various plant species. It also increases starch accumulation by inhibiting the translocation of photo-assimilates from the leaves. However, *Lactuca sativa* plants grown under red LEDs showed lower rates of photosynthesis once light intensity decreased [10]. Similar results of the reduced rate of photosynthesis under low light intensity and red LEDs was reported for rice [11] and wheat [12]. Such results may suggest that vulnerability to a lower photosynthetic rate might be linked with changes in multiprotein complexes such as photosystem I (PSI) and photosystem II (PSII) [10, 13] or it could be attributed to lower leaf nitrogen content because of altered chlorophyll and carotenoid contents [14].

Lettuce plants grown under blue LEDs and high light intensity, displayed a higher net photosynthetic rate which could be an indicative of the fact that the plants had a pronounced acclimation of photosystems for CO_2_ fixation [10]. A blue light supplementation also led an increase of photosynthetic activity of *Convallaria majalis* and strawberry plants [15, 16]. Research conducted on spinach plants grown under blue light-deficient conditions revealed that the CO_2_ assimilation rate and quantum efficiency of PSII (ΦPSII) increased, while the efficiency of light capture (Fv’/Fm’) decreased [17].

Considering the effects of green light, the poor absorption of this wavelength by plant leaves is well explained through the absorption spectra of chlorophylls or other plant pigments extracted from green leaves [18]. The work of Wu et al. reported a higher photosynthetic efficiency in tomato plants grown under green light in comparison to plants grown under yellow light [19]. This is in line with the physical laws of light quanta, with shorter wavelength photons possessing more energy and therefore exciting more chlorophyll molecules to an unproductive singlet state. Similarly, when lettuce plants were grown under high-intensity (≥PPF 300 μmol m^-2^ s^-1^) green LEDs, they showed normal development like plants grown under a fluorescent lamp which indicated the fact that green light at higher intensities is available to the plants for morphogenesis and photosynthesis while green light of low intensity might penetrate the leaf insufficiently for morphogenesis or photosynthesis [20].

Recent work conducted by Liu and van Iersel [21] showed that at low PPFD, red light has a higher quantum yield than both blue and green light, which is consistent with the McCree curve. As photosynthetic photon flux density (PPFD) increased, quantum yield of green light decreased more slowly than that of red and blue light. As a result, the quantum yield of green light gradually surpassed that of blue light and was similar to that of red light at high PPFD i.e., under low PPFD red light the quantum yield was higher under red (peak λ 653 nm) than blue (peak λ 446 nm) and green (peak λ 523 nm), while under high PPFD, the application of green color caused the higher observed quantum yield (Green>red>blue).

Chlorophyll a fluorescence measurement is widely used in studies of photosynthetic processes [22, 23], monitoring of plants abiotic stress responses see [24], nutrient status of plants [23, 25–30], plant breeding to select plants of high photosynthetic capacity [31, 32], or in postharvest quality studies or assessments of horticultural and other crop plants [33].Evaluation of the LEDs quality that produces the best vigorous of tomato seedlings based on its physiological performance was the main aim of this work. This experiment was designed on the hypothesis that the response of the photosynthetic apparatus of tomato cultivars (*Solanum lycopersicum var*. Gangmu No.1) and (*Solanum lycopersicum var*. Millennium) and their growth parameters will be varied base on the characteristics of the applied light.

## 2 Materials and methods

### 2.1 Growth conditions and plants cultivation

**Fig 1:**
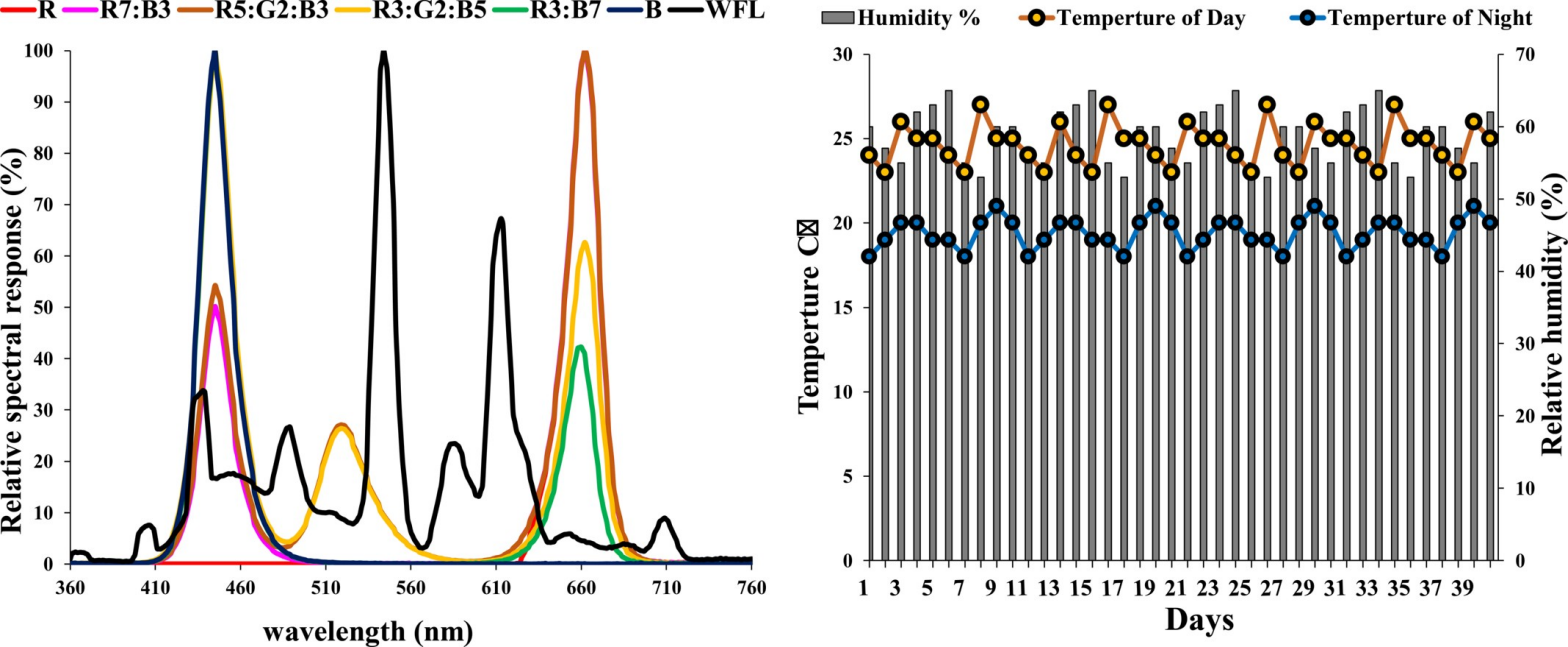
Relative spectral response (%) of the treatments in chambers and environmental conditions.

The experiment was conducted in growth chambers equipped with various LEDs (with possibility to control light intensity and quality in each compartment, separately) at Fujian Agriculture and Forestry University, Fujian, China. The experimental design included seven experimental variants. Each variant had 3 chambers as replicates. Single chamber dimensions were 60 x 60 x 60 cm. The day/night temperatures were 25±2 ˚C and 19±2 ˚C, respectively. The average relative humidity varied in the growth chambers during the experiment and was 55± 5% (Fig 1).

Seeds of two cultivars of tomato (*Solanum lycopersicum*) Gangmu No.1 and Millennium were sown in 32-cells plug trays (28 cm width × 54 cm length × 8 cm height., Luoxi Plastic Products Co., Shandong, China). Two trays were placed in each growth chamber (1 tray contained 32-cells for each cultivar). The trays were filled with commercial growth substrate (N1:P1:K1 ≥ 3%, Organic matter ≥ 45%, pH 5.5−6.5, Jiangping Enterprise Co., Fujian, China).

One week after sowing, seedlings started to receive fertilization based on water-soluble fertilizers (compound fertilizers "N20: P20: K20+TE", Ruierkang Co., Russia) and Stimufol Amino (compound fertilizers “N 25%, P 16%, K 12%, Amino acids 2%, Bo 0.044%, Fe 0.17%, Mo 0.001%, Zn 0.03%, Cu 0.085, Co 0.01%, Mg 0.02%, Mn 0.085% and EDTA” Shoura Co., Egypt.). Seedlings also were irrigated two times per week to keep the optimum level of water content in the growth substrate.

Seedlings were grown in growth chambers under 100± 2 μmol photons m^-2^ s^-1^ light intensity for 12 hours per day. The applied LEDs spectra were: R (red peak wavelength λp (662 nm)), R7:B3 (red 70 + blue 30 peak wavelength λp (662 nm)), R5:G2:B3 (red 50+ green 20+ blue 30 peak wavelength λp (662 nm)), R3:G2:B5 (red 30 + green 20 + blue 50 peak wavelength λp (445 nm)), R3:B7 (red 30+ blue 70 peak wavelength λp (445 nm)), B (blue peak wavelength λp (445 nm)), and WFL (white florescent light peak wavelength λp (544 nm)), as control.

After 21 days, seedlings were transplanted into plastic pots (D 7 cm × H 10 cm; Luoxi Plastic Products Co., Shandong, China) that was filled with the same commercial growth substrate as it was in the 32-cell plug trays to allow the root system to grow, freely.

### 2.2 Plant growth characteristics

Data of growth parameters were collected on the 40^th^ day after sowing (DAS). Plant height (cm) was measured manually. Stem diameter (mm) was measured using a digital Vernier caliper. Leaf area (cm^2^) was determined according to Pandey and Singh [34] method, and total leaf area (cm^2^) was calculated by the number of leaves × leaf area. The fresh plants were placed in Petri dishes without cover and transferred to a drying oven at 75°C for at least for 48 h to obtain the dry weight. Dry matter was weighed using an electronic balance (±0.0001 g). Dry matter content percentage was calculated by the use of the following equation:

Drymattercontent%=(Plantdryweight/Plantfreshweight)×100

### 2.3 Chlorophyll *a* fluorescence measurements

Chlorophyll fluorescence signals were measured on the 40^th^ day after sowing (DAS) using PAM-2500 chlorophyll fluorometer (Heinz Walz GmbH, Effeltrich, Germany). Measurements were done on the fourth leaf (fully developed leaf), selected from the top of each plant. Measurements were done on 4 leaves from 4 different plants per experimental variant (4 measurements × 4 leaves = 16 replicants). The leaf area of the standard measuring head according to the size of the clip was 1.3 cm^2^.

Two types of chlorophyll *a* fluorescence induction kinetics were managed. The first measurement was based on the application of saturation pulse from red LEDs (8000 μmolm^-2^ s^-1^; 300 ms duration) to determine the minimum (Fo) and maximum chlorophyll fluorescence (Fm) after dark adaptation of the samples for 30 min (by the use of the special leaf clips). After that, 14 pulses with the same light intensity were applied with 20 seconds intervals. The first pulse was applied after 40 seconds from first measurements. Data are shown in Fig 3.

The second type of chlorophyll fluorescence induction kinetics was the performance of rapid light curve (RLC). The light intensity gradient of the RLC was 0, 1, 30, 63, 100, 140, 197, 270, 362, 618, 784, and 1159 μmol photons m^-2^ s^-1^. The duration of each light intensity was 20 s. Data are shown in Fig 4.

Under both above mentioned chlorophyll fluorescence measurement protocols the effective quantum yield of PSII photochemistry Y[II], the quantum yield of non-regulatory energy dissipation [Y(NO) = Fs/Fm] and the quantum yield of regulatory energy dissipation [Y[NPQ] = 1- Y[II]—Y(NO)] were assessed. Non-photochemical quenching (NPQ), photochemical quenching (qP), and the electron transport rate (ETR) were evaluated [35].

### 2.4 Data analysis

The data were subjected to two-way ANOVA (LEDs x Cultivar). The comparison between treatment means were done using Duncan’s multiple range test when *P ≤ 0*.*05* [36]. Pearson’s correlation coefficient was executed using SPSS statistical software package version 16.0 (SPSS Inc., Chicago, IL, USA).

## 3 Results

### 3.1 Plant growth characteristics

**Fig 2:**
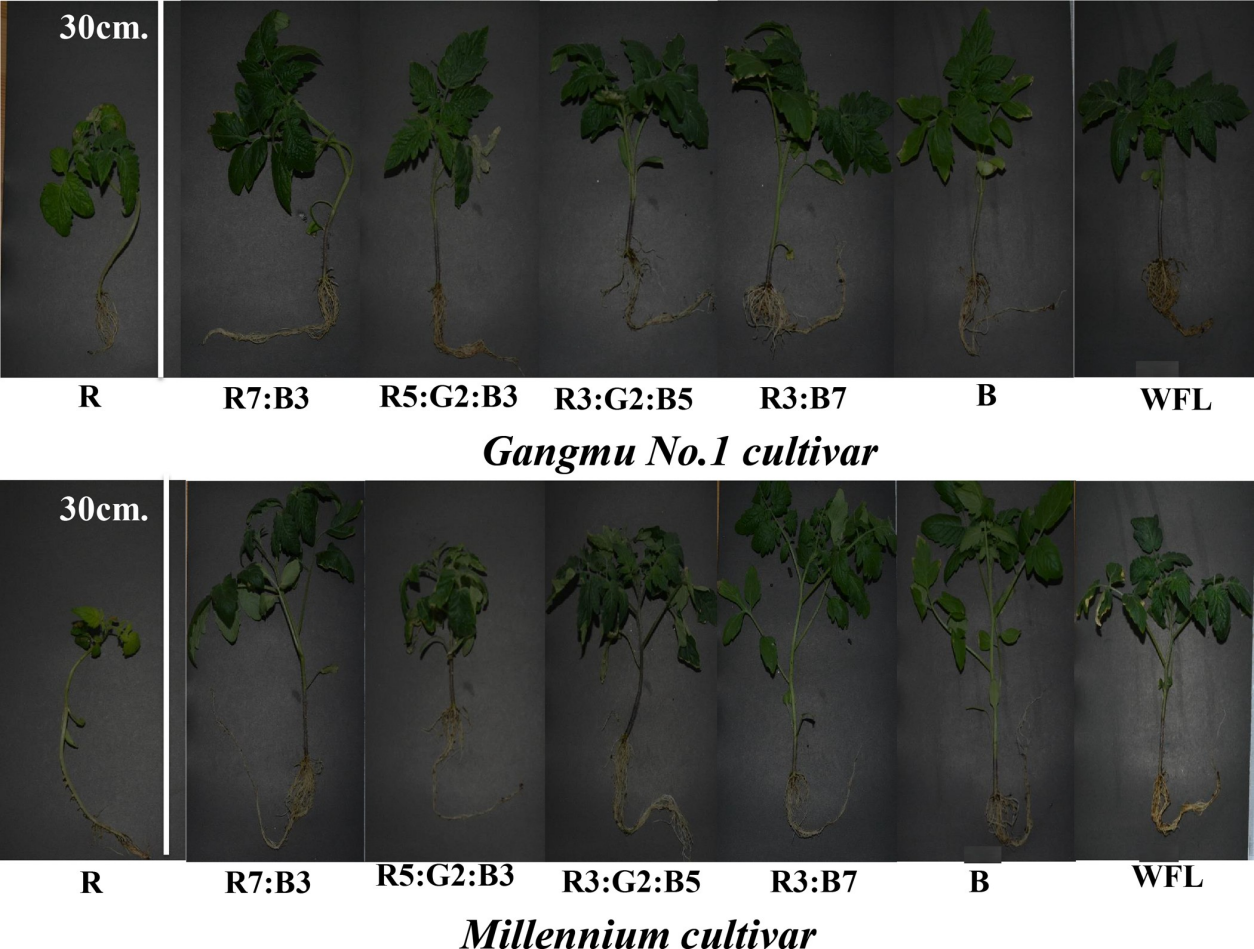
Tomato seedlings grown 40 days under different treatments of LED light.

Light quality with LEDs had significant effects on tomato seedlings’ morphological appearances (Table 1 and Fig 2). The plants of Millennium cultivar exhibited more height (21.79 cm) than the plants of Gangmu No.1 (20.30 cm), indicating positive response of Millennium in terms of biomass accumulation. On other hand, R7:B3 proved best (28.5% efficient than WFL) among all other applied combinations of light with respect to the plant height of tomato seedlings. Plant height of tomato seedlings under the influence of R7:B3 was recorded maximum in both cultivars (24.33 cm ‘Gangmu No.1’, 26.67 ‘Millennium’). By DMR statistical test, it has been evaluated that R3:G2:B5 was slight but non-significantly different than WFL (control), while R3:G2:B5 was observed to be 1.14-fold lower than WFL. There was non-significant difference among both cultivars with respect to stem diameter. The R7:B3 proved best (32.71% efficient than WFL) among all other applied combinations of light in terms of stem diameter. Stem diameter of tomato seedlings under the influence of R3:B7 was recorded maximum in both cultivars (3.26 mm ‘Gangmu No.1’, 3.75 mm ‘Millennium’). In case of area of total leaves per plant, the difference among both cultivars was comparable. The R7:B3 treatment exhibiting maximum total leaves area (44.92% more than WFL) among all other light combinations, was most promising LED application on seedlings of tomato. The maximum area of leaves was exhibited by the plants of both cultivars exposed to R3:B7 (194.75 cm^2^ ‘Gangmu No.1’, 231.71 cm^2^ ‘Millennium’). Millennium expressed 2.8% increase in plant diameter as compared to Gangmu. The R5:G2:B3 treatment was comparable to WFL. Dry matter content of tomato seedlings of Gangmu No.1 under the influence of R5:G2:B3 was recorded maximum (13.58%), while Millennium was greatly influenced by WFL (13.34%).

**Table 1:** Effect of LED light quality on plant growth characteristics of tomato seedlings Gangmu No.1 and Millennium cultivars.

Treatments	Plant height (cm)			Stem diameter (mm)			Total Leaves area (cm^2^)			Dry matter content %		
	Gangmu No.1	Millennium	LEDs Mean	Gangmu No.1	Millennium	LEDs Mean	Gangmu No.1	Millennium	LEDs Mean	Gangmu No.1	Millennium	LEDs Mean
**R**	**21.07 b**	**22.00 b**	**21.53 b**	**2.85 ab**	**2.66 b**	**2.76 bc**	**123.67 d**	**71.33d**	**97.50 d**	**8.42 e**	**10.93 d**	**9.68 d**
**R7:B3**	**24.33 a**	**26.67 a**	**25.5 a**	**3.05 ab**	**3.57 a**	**3.31 a**	**194.75 a**	**231.71 a**	**213.23 a**	**11.81c**	**12.47 b**	**12.14 b**
**R5:G2:B3**	**17.67 c**	**17.00 d**	**17.34d**	**2.96 ab**	**2.70 b**	**2.83 bc**	**117.33 d**	**85.13 cd**	**101.23 d**	**13.58 a**	**12.49 b**	**13.04 a**
**R3:G2:B5**	**19.50 bc**	**19.00 cd**	**19.25 c**	**3.10 ab**	**3.58 a**	**3.34 a**	**184.45 ab**	**141.96 bc**	**163.21 bc**	**13.00 ab**	**12.00 bc**	**12.5 b**
**R3:B7**	**21.00 b**	**23.00 b**	**22.00 b**	**3.26 a**	**3.75 a**	**3.26 a**	**176.27 a-c**	**203.81 ab**	**190.04 ab**	**12.36 bc**	**12.07 b**	**12.22 b**
**B**	**20.50 b**	**23.17 b**	**21.84 b**	**2.95 ab**	**3.24 ab**	**3.10 ab**	**143.27 b-d**	**191.92 ab**	**167.60 bc**	**10.56 d**	**11.40 d**	**10.98 c**
**WFL**	**18.00 c**	**21.67 bc**	**19.84 c**	**2.63 b**	**2.69 b**	**2.66 c**	**134.87 cd**	**159.41 b**	**147.14 c**	**12.68 b**	**13.34 a**	**13.01 a**
**Cultivars mean**	**20.30 b**	**21.79 a**		**2.97 a**	**3.07 a**		**153.52 a**	**155.04 a**		**11.77 b**	**12.10 a**	

Values are means of three replicates. Different letters in the same column indicate significant differences according to Duncan’s multiple range test at P≤ 0.05. Where R = Red light 100%, R7:B3 = Red70%+ Blue30%, R5:G2:B3 = Red50%+ Green20%+ Blue30%, R3:G2:B5 = Red30%+ Green20%+ Blue50%, R3:B7 = Red70%+ Blue30%, B = Blue light 100%, WFL = White Fluorescent Lamps100%.

### 3.2 Chlorophyll *a* fluorescence measurements

#### 3.2.1 Measurements chlorophyll *a* fluorescence induction kinetics of the dark-acclimated tomato plants

**Fig 3:**
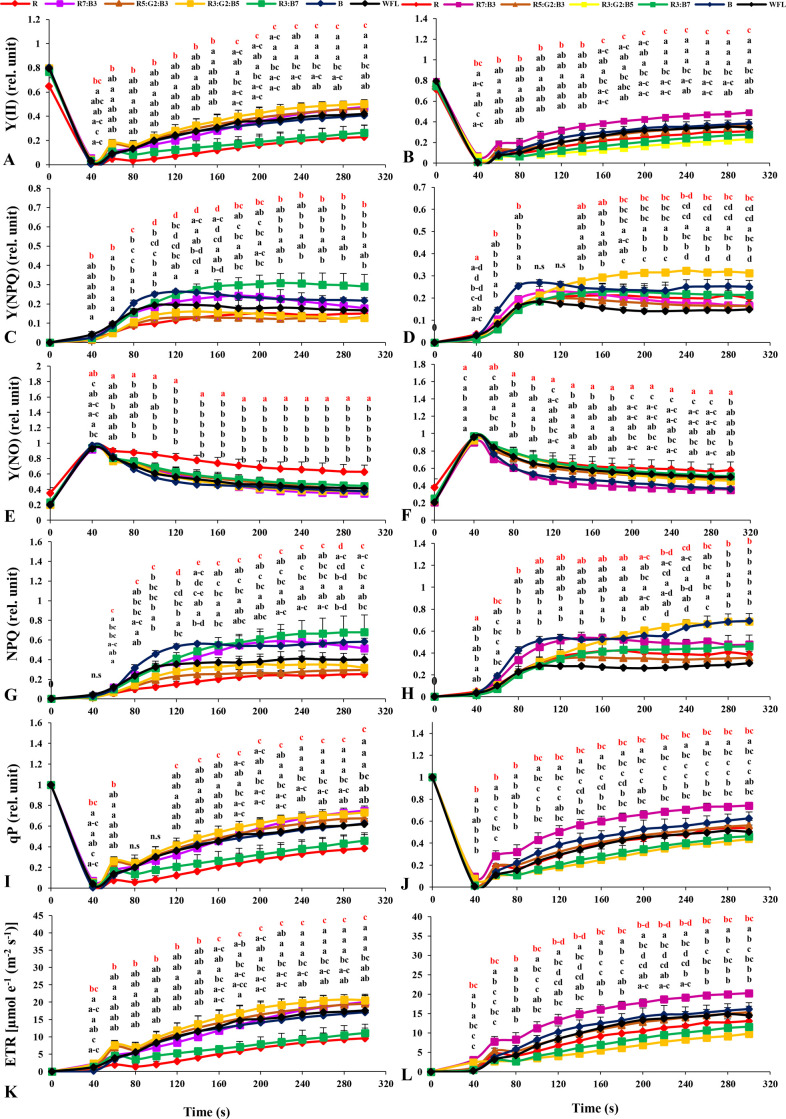
Effects of LED light quality on chlorophyll *a* fluorescence induction kinetics of the dark-acclimated tomato plants. A and B: effective quantum yield of PSII photochemistry Y(II), C and D: quantum yield of regulatory energy dissipation in PSII Y(NPQ), E and F: quantum yield of non-regulated energy dissipation in PSII Y(NO), G and H: non-photochemical quenching (NPQ), I and J: photochemical quenching coefficient (qP), and K and L: electron transport rate (ETR) in tomato leaves. Gangmu No.1 (A,C,E,G,I,K) and Millennium (B,D,F,H,J,L). Line markers on the curves indicate to the average± standard error (P≤0.05, n = 4). Sort significance letters from top to bottom according to the treatments (R, R7:B3, R5:G2:B3, R3:G2:B5, R3:B7, B, WFL). Where R = Red light 100%, R7:B3 = Red70%+ Blue30%, R5:G2:B3 = Red50%+Green20%+ Blue30%, R3:G2:B5 = Red30%+Green20%+ Blue50%, R3:B7 = Red70%+ Blue30%, B = Blue light 100%, WFL = White Fluorescent Lamps100%.

Kinetic measurements (5 min in total) were performed on the dark-acclimated leaves, and the results are shown in Fig 3. Dark acclimation allowed the opening of PSII reaction centers, oxidation of the electron transport chain, relaxation of photoprotective mechanisms (Xanthophyll Cycle), and depletion of the trans-thylakoid gradient. The effective quantum yield of PSII photochemistry Y[II] slowly began to increase with continued exposure to higher light intensity in both varieties. In the cultivar Gangmu No.1, the value of Y[II] was highest under R3:G2:B5 during 60-140s of the measurement time (Fig 3A), while it was highest in the cultivar Millennium under R7:B3 at 40-300s (Fig 3B).

With increasing light intensity, the quantum yield of regulated energy dissipation in PSII [Y[NPQ]] improved rapidly in both cultivars. The [Y[NPQ]] in cv. Gangmu No.1 was highest under treatments B, WFL, and R3:B7 at 60–100, 120–140, and 160-300s, respectively (Fig 3C). On the other hand, in cv. Millennium, it was highest under treatments B and R3:G2:B5 at 60–80 and 140-300s, respectively (Fig 3D).

The quantum yield of non-regulated energy dissipation in PSII [Y(NO)] increased rapidly at the onset of light exposure and decreased directly after 40s, with an increase in the time period for all light qualities in both cultivars. The [Y(NO)] in cultivar Gangmu No.1 was highest under R treatment at 60-300s (Fig 3E), while in cv. Millennium it was highest under R treatment at 120-300s (Fig 3F).

Non-photochemical quenching (NPQ) increased rapidly with increasing time in both varieties at all light qualities. The (NPQ) in cultivar Gangmu No.1 was highest under B and R3:B7 light treatments at 40-160s and R7:B3, R3:B7 and B treatments at 160-300s (Fig 3G), while in cv. Millennium this parameter was highest under B at 40-120s, R7:B3 at 140-160s, R3:G2:B5 at 180-280s and then B treatment at 300s (Fig 3H).

The photochemical quenching coefficient (qP) decreased directly with the stable light intensity of 100 μmol photons m^-2^ s^-1^ at all light qualities in both cvs. and then increased rapidly with continued light exposure at all light qualities in both cvs. The qP in cv. Gangmu No.1 was highest under R3:G2:B5, R5:G2:B3, and B at 80-300s (Fig 3I), while in cv. Millennium it was highest under B and R7:B3 at 80-300s (Fig 3J).

The electron transport rate of PSII (ETR) increased rapidly with increasing exposure time in all light qualities in both tested cultivars. The ETR developed best under R3:G2:B5 in cv. Gangmu No.1 (at 40-160s and then at 200-300s) (Fig 3K), while the highest value was under R7:B3 in cv. Millennium at 40-300s (Fig 3L).

#### 3.2.2 Effects of LED light quality on RLC of the light-acclimated tomato plants

**Fig 4:**
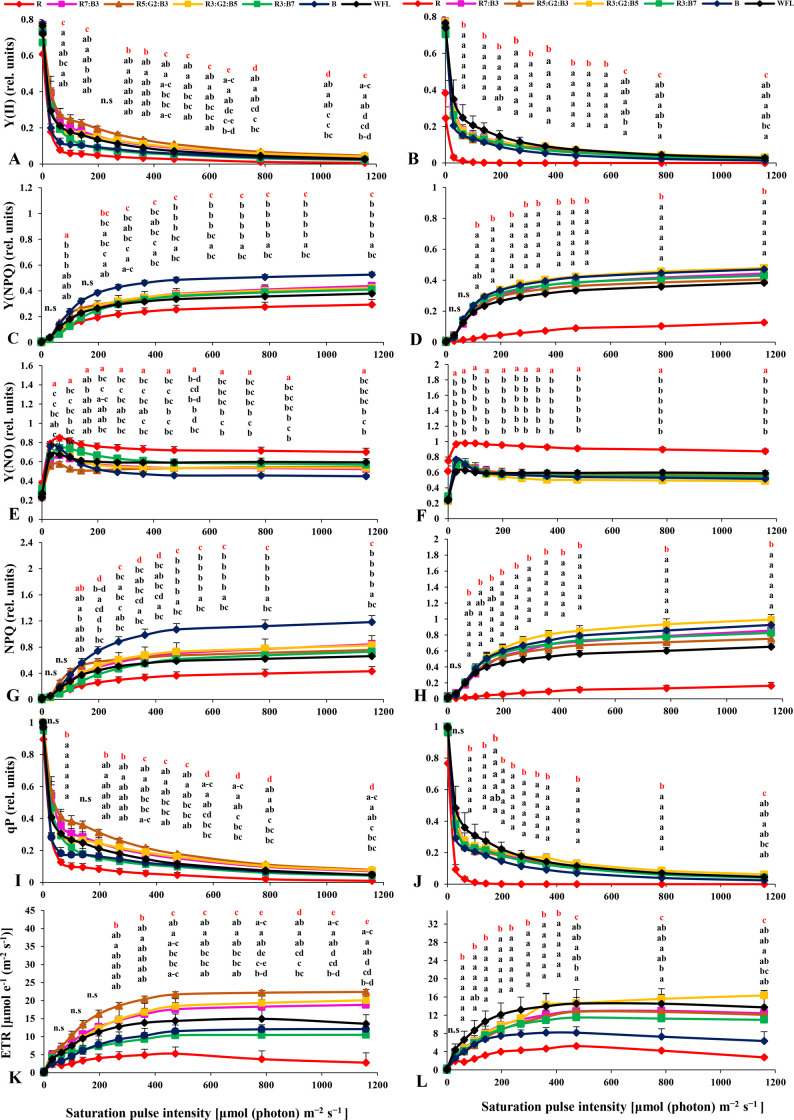
Effects of LED light quality on RLC of the light-acclimated tomato plants. A and B: effective quantum yield of PSII photochemistry Y(II), C and D: quantum yield of regulatory energy dissipation in PSII Y(NPQ), E and F: quantum yield of non-regulated energy dissipation in PSII Y(NO), G and H: non-photochemical quenching (NPQ), I and J: photochemical quenching coefficient (qP), and K and L: electron transport rate (ETR) in tomato leaves. Gangmu No.1 (A,C,E,G,I,K) and Millennium (B,D,F,H,J,L). Line markers on the curves indicate to the average± standard error (P≤0.05, n = 4). Sort significance letters from top to bottom according to the treatments (R, R7:B3, R5:G2:B3, R3:G2:B5, R3:B7, B, WFL). Where R = Red light 100%, R7:B3 = Red70%+ Blue30%, R5:G2:B3 = Red50%+Green20%+ Blue30%, R3:G2:B5 = Red30%+Green20%+ Blue50%, R3:B7 = Red70%+ Blue30%, B = Blue light 100%, WFL = White Fluorescent Lamps100%.

Rapid light curves (RLCs) of light-acclimated photosynthetic quantum yields for PSII were measured. The LED-induced differences in the effective quantum yield of PSII photochemistry Y[II] manifested mainly in the range of 50–500 light intensities. In this range, the highest Y(II) values for R5:G2:B3 were obtained in Gangmu No. 1, while hardly any significant differences were observed in Millennium. In the latter, WFL showed the highest Y(II) (Fig 4A and 4B). It gradually decreased with increasing light intensity in all light quality treatments. The Y[II] under R5:G2:B3 and R3:G2:B5 was significantly higher than the others for cv. Gangmu No.1 at almost all light intensities (Fig 4A), while under WFL it was significantly higher than the other treatments for cv. Millennium at almost all light intensities (Fig 4B).

The quantum yield of regulated energy dissipation in PSII [Y (NPQ)] was highest under B in Gangmu No 1, but significant differences were hardly observed in cv. Millennium. The blue light (B) provided the highest [Y (NPQ)] in both tested varieties (Fig 4C and 4D). The quantum yield of nonregulated energy dissipation in PSII [Y(NO)] had the highest value under R in both cultivars (Fig 4E and 4F). It increased rapidly with initial light exposure and stabilized after a slight decrease. However, it increased with increasing light intensity up to 60 μmol photons m^-2^ s^-1^ at all light qualities in both cultivars. The Y(NO) was highest under the R treatment in both cultivars at all light intensities (Fig 4E and 4F).

Non photochemical quenching (NPQ) had the highest value under B and R3:G2:B5 in both cultivars studied (Fig 3G and 3H). The (NPQ) gradually increased with increasing light intensity under all light qualities in both cultivars. The NPQ was significantly higher under B and R3:G2:B5 treatments compared to WFL at almost all light intensities in both cultivars studied (Fig 4G and 4H).

The photochemical quenching coefficient (qP) had the highest value under treatments R5:G2:B3 and WFL in both cultivars (Fig 4I and 4J). It gradually decreased with increasing light intensity under all tested light qualities in both cultivars. The qP was significantly lower under R, R3:B7 and B compared to WFL at almost all light intensities in both cultivars (Fig 4I and 4J).

The electron transfer rate (ETR) of PSII was better under R5:G2:B3 in cultivar Gangmu No.1 (Fig 4K), while it was best under WFL in cultivar Millennium before the light intensity reached 270 μmol photons m^-2^ s^-1^ and R3:G2:B5 after the light intensity reached 270 μmol m^-2^ s^-1^ (Fig 4L). The ETR increased with increasing light intensity and became constant when the light intensity reached 473, 618, 748, and 1159 μmol photons m^-2^ s^-1^, respectively, depending on the treatments, except for R, which decreased after reaching 473 μmol photons m^-2^ s^-1^ in both varieties. The ETR the plants treated with R5:G2:B3 light was significantly higher in the cultivar Gangmu No.1 compared with the other treatments (Fig 4K), while it was statistically similar in all other treatments at all light intensities, except for R, where it was lower in the cultivar Millennium (Fig 4L).

#### 3.2.3 Correlations between morphological and physiological parameters

Pearson’s correlation [37] was carried out among the morphological and physiological parameters observed in this study (Table 2). A significant positive correlation between Y[II] and qP (R^2^ = 0.899), Y[II] and ETR (R^2^ = 0.871), qP and ETR (R^2^ = 0.966), NPQ and Y[NPQ] (R^2^ = 0.791) were observed in cv. Gangmu No.1. Only two correlations were found between morphological and physiological tested parameters i.e., NPQ and stem diameter (SD) (R^2^ = 0.758) or total leaves area (TLA) (R^2^ = 0.788). In the case of cultivar ‘Millennium’, a positive significant relationship was found between qP and ETR (R^2^ = 0.945), Y[II] and ETR (R^2^ = 0.959), and NPQ and Y[NPQ] (R^2^ = 0.9113). Only one correlation between morphological and physiological parameters was found i.e., qP and total leaves area (TLA) (R^2^ = 0.426), (Table 2).

**Table 2:** Correlation coefficient evaluation between morphological and physiological parameters.

		PH	SD	TLA	DM	Y[II]	Y[NPQ]	Y[N0]	NPQ	qP	ETR
**Gangmu No.1**	PH	1									
	SD	0.423	1								
	TLA	0.644	0.656	1							
	DM	-0.425	0.205	0.252							
	Y[II]	-0.152	-0.052	0.278	0.664	1					
	Y[NPQ]	0.328	0.485	0.310	-0.072	-0.502	1				
	Y[N0]	-0.045	-0.234	-0.523	-0.708	-0.824*	-0.077	1			
	NPQ	0.521	0.758*	0.788*	0.156	-0.053	0.791*	-0.454	1		
	qP	-0.03	-0.342	0.121	0.406	0.900**	-0.471	-0.740	-0.167	1	
	ETR	-0.273	-0.467	-0.084	0.462	0.871*	-0.501	-0.686	-0.297	0.967**	1
**Millennium**	PH	1									
	SD	0.426	1								
	TLA	0.747	0.749	1							
	DM	-0.081	-0.06	0.277	1						
	Y[II]	0.571	-0.071	0.410	0.235	1					
	Y[NPQ]	-0.200	0.512	0.038	-0.499	-0.599	1				
	Y[N0]	-0.407	-0.465	-0.679	-0.154	-0.647	-0.135	1			
	NPQ	0.117	0.714	0.320	-0.564	-0.292	0.911**	-0.405	1		
	qP	0.557	0.085	0.426**	-0.046	0.926	-0.391	-0.711	-0.023	1	
	ETR	0.474	-0.036	0.427	0.255	0.959**	-0.605	-0.656	-0.281	0.945**	1

* = Correlation is significant at the *P≤ 0*.*05* level,

** = Correlation is significant at the *P≤ 0*.*01* level, by using Pearson correlation coefficients. Where PH = Plant height, SD = Stem diameter, TLA = Total leave area, and DM = Dry matter contents%.

## 4 Discussion

In general, not only the performance of the photosynthetic apparatus of plants is strongly influenced by the quality of light, but also their morphological and physiological properties [38]. In this work, we have tried to understand how the photosynthetic efficiency of plants and their growth are affected by different light quality and quantity.

The process of photosynthesis requires light capture and utilization. Leaves are affected by the directional quality of incoming light. They are not simple light absorbing objects and cellular anatomy affects the direction of light entering the mesophyll [39]. Stimulation of growth by different wavelengths is already known, e.g., how wavelengths can affect plant growth through light signal transduction pathways [40, 41]. During the transition from dark to light, the rate of hypocotyl elongation is determined by the integration of light signals perceived through the phototropin, cryptochrome, and phytochrome signaling pathways. For example, blue light is absorbed by cryptochrome and phototrophic photoreceptors [40, 41] and can inhibit shoot elongation [42].

Our results contradict studies reporting inhibition of seedling growth under mixed R and B lights compared to monochromatic light [43–46]. Seedlings of the two tested cultivars (Gangmu No.1 and Millennium) showed maximum plant height, stem diameter and total leaf area when grown under R7:B3, indicating that this spectral composition is most suitable for their growth (Table 1). Similar results were obtained by Yang, He [47] in pepper seedlings and Kim and Hwang [48] in tomato seedlings. They observed that a mixture of red and blue LED light had a beneficial effect on total leaf area and plant height. Thus, the observed stimulation of plant elongation in our experiment could be due to the reduction in the ratio of blue to red light [49].

Light quality affects photochemical reactions due to the difference in luminous efficiency of the various photosynthetic pigments [50–52]. In addition, a certain spectrum of solar radiation can damage the photosystems, especially PSII, and cause further photoinhibition [53–55]. Light quality can directly affect the photochemical response in short-term illumination. It can also indirectly affect photosynthetic electron transport by regulating several biochemical processes such as endogenous hormone balance and metabolic responses [56]. In dark-acclimated leaves of tomato, the parameter Y[II ] increased consistently with elapsed time (Fig 3A and 3B). This was mainly due to the activation of enzymes involved in carbon metabolism and stomata opening [57]. On the other hand, Y[II] decreased while photosynthetically active radiation (PAR) increased (0, 30, 63, 100, 140, 197, 362, 618, 784, and 1159 μmol photons m^-2^ s^-1^) (Fig 4A and 4B). This may be due to excessive reduction of PSII subunits. The lower Y[II] observed under red light in cv. Gangmu No.1. This may be due to the fact that red light efficiently induces inactivation of the PSII reaction center in Tris-treated thylakoid membranes [58].

The fraction of energy dissipated in the form of heat regulates the NPQ photoprotective mechanism Y[NPQ] and was larger when the value of Y[II] approached zero at high quantum flux densities. This may indicate a high photoprotective capacity [59]. The results of our study showed that the values of Y[NPQ] were significantly different between treatments under dark and light acclimation conditions (Figs 3 and 4C and 4D).

A greater proportion of energy dissipated passively in the form of heat and fluorescence, mainly by closed PSII reaction centers (Y(NO)), reflects the inability of a plant to protect itself from damage caused by excessive illumination [59]. Our results showed a decrease in Y(NO) under dark and light acclimation conditions (Figs 3 and 4E and 4F). An inability of seedlings to protect against excessive illumination was observed in both cultivars under R light conditions (Figs 3 and 4E and 4F). Interestingly, Hamdani, Khan [60] recently showed that significant down-regulation of the catalase (CAT) and ascorbate peroxidase (APX) transcripts was observed in rice treated with both red and blue light compared with those treated with white light. Therefore, the weakened antioxidant system and accumulated reactive oxygen species in tomato plants grown under red light regimes may have played a significant role in the observed changes in Y(NPQ) and Y(NO).

To reduce light damage, plants have evolved several protective mechanisms, including nonphotochemical quenching (NPQ), which quenches the excitation of the chlorophyll molecule within the light-collecting antennae of PSII by converting the excitation energy into thermal energy that can then be emitted [61, 62]. Our results showed that the value of NPQ under dark acclimation and light acclimation increased continuously with increasing time and light intensity, respectively (Figs 3 and 4G and 4H). According to the NPQ model proposed by Zaks, Amarnath [63] and Chávez-Arias, Gómez-Caro [64]tomato leaves are often unable to use all the light absorbed by their photosynthetic pigments for CO_2_ fixation. The limited CO_2_ fixation limits photosynthetic electron transport, which in turn limits the performance of photosystem I and II reaction centers. In the case of PSII, these results lead to reactions that generate deleterious oxygen [65] as well as damage to the reaction center [66] and membranes [67, 68].

Photochemical quenching (qP) is used as a parameter for estimating the fraction of PSII centers in open states based on a puddle model for the photosynthetic unit [69]. qP value increased under dark-acclimation of tomato leaves (Fig 3I and 3J), indicating an increase of the active reaction centers over the elapsed time. These results are in agreement with Wang et al. [70]) work, where it was found that the qP of *Dendrobium candidum* plantlets under red and blue compound light were higher than monochrome red light. On the other hand, qP under light-acclimation was observed to be decrease with increase in light intensity (0, 30, 63, 100, 140, 197, 362, 618, 784, and 1159 μmol photons m^-2^ s^-1^) and also decreased with time (Fig 4I and 4J).

Electron transport rate (ETR) is also a light-acclimated parameter that is directly correlated to Y[II] by the equation ETR = PAR x Y(II) x Abs-factor (absorbance of photons by photosynthetic pigments) x ratio of absorbed photons by PSII and by photosynthetic pigments [71]. In present study, ETR was continuously increasing in time or increasing light intensity (Figs 3 and 4K and 4L). This was denoted in both cultivars and all light treatments. However, the increment of ETR value in time was continues up to 300s (Fig 3K and 3L), while it stopped to increase after light intensities of ca. 400 μmol photons m^-2^ s^-1^ (Fig 4K and 4L) which means that the latter was the maximum light for growing these plants, nevertheless of the applied light quality.

Several studies showed that light quality has significant effect on plants growth and photosynthetic efficiency of plants. Leaves of *Cunninghamia lanceolata* exposed to light (72.7% red+ 9.1% blue+ 9.1% purple+ 9.1% green) generated more roots and presented higher chlorophyll, Fv/Fo, Y (II), qP, and ETRII than white light (12.7% red+ 3.9% blue+ 83.4% green) during photoperiods of 8 and 16 h [72]. The exposure to R3:G2:B5 in Gangmu No.1 and R7:B3 in Millennium cultivar might have led to higher cyclic electron transport and consequently to acidification of thylakoid lumen and higher NPQ, avoiding that these electrons are used for photosynthesis [73]. On the other hand, green light can penetrate deeper in the leaf than red or blue light [18, 74], thus leading to an increase of ETR and consequently to a stronger activation of inner leaf photosynthesis and carboxylation activity of RuBisCo [18]. The work conducted by Folta [75] showed that green light promoted early stem elongation that antagonizes growth inhibition of Arabidopsis seedlings. Liu and van Iersel [21] also showed that plants can exploit from green light but only under high light intensities.

Measurements of chlorophyll *a*, chlorophyll *b*, carotenoids, nitrate, soluble protein, and soluble sugar showed significant difference among the treatments (unpublished data). Chlorophyll *a*, chlorophyll *a+b*, and carotenoids contents were lower under red light in both cultivars. The reason might be that the pure red light did not give enough absorbed light energy causing low Fv/Fm, Y(II), Y(NPQ), ETR, and qP. The high Y(NO) suggests that the function of PSII is not well operated under red light solely, and it might alter the structure of PSII. Chlorophyll a had the highest value under R3:G2:B5 in Gangmu No.1 and under R7:B3 in Millennium cultivar. The reason could be the difference in the effective photochemical quantum yield of photosystem II value resulting from the different optical spectra’s effect between the different cultivars. Our results showed that the effect of the optical spectra differs in the different genotypes within the same plant species. Ouzounis et al. [76] examined the effect of 100% red and 88% red/12% blue on nine tomato genotypes and reported that the combination of blue and red LED lighting increased chlorophyll and flavonols contents in three genotypes.

Considering that tomato leaves of both cultivars treated with red light have increased Y(NO), we think that these plants have impaired antioxidant capacity compared to plants grown under other light regimes, leading to an increase in reactive oxygen species. reported that significant down-regulation of transcripts of catalase (CAT) and ascorbate peroxidase (APX) was observed in rice treated with both red and blue light compared to plants treated with white light. Therefore, the weakened antioxidant system and, accordingly, the accumulated reactive oxygen species, as represented here by H_2_O_2_, in tomato plants grown under red light regimes may have played a role in the observed changes in Y(II), Y(NO), and Y(NPQ).

Our results suggest that the responses of the photosynthetic apparatus to light regimes are cultivation-dependent [77]. In the case of cv. Gangmu No.1, the combinations R3:G2:B5 and R7:B3 were the best light regimes for the photosynthetic process, while in the case of cv. "Millennium" it was R7:B3 and WFL.

The only logical/explanatory significant relationship between morphological and physiological test parameters to be highlighted from our work was between qP and total leaves area (R^2^ = 0.426) in cv. Millennium. This indicates the weak relationship between the growth parameters tested in this work and the photosynthetic efficiency of the plants and that such relationships are cultivation dependent. It appears that this is because different light regimes affect plant photosynthetic efficiency rather than growth parameters.

## 5 Conclusions

The photosynthetic efficiency of the plants of the two tomato cultivars tested was significantly altered as a function of the light regime applied. This was observed under both types of chlorophyll fluorescence applied measurements. However, we found that the response of the photosynthetic apparatus of tomato plants to light regimes is cultivar dependent. Variations among these traits suggest that the linking between morphological and physiological traits is insufficient to understand the effect of the optical spectrum of light on plant growth. Therefore, it is necessary to perform molecular analyses and link them to morphological and physiological traits to learn more about the mechanisms of the effect of LED light on seedling growth.
